# A Loose Parts Randomized Controlled Trial to Promote Active Outdoor Play in Preschool-aged Children: Physical Literacy in the Early Years (PLEY) Project

**DOI:** 10.3390/mps2020027

**Published:** 2019-04-04

**Authors:** Natalie E. Houser, Jane Cawley, Angela M. Kolen, Daniel Rainham, Laurene Rehman, Joan Turner, Sara F. L. Kirk, Michelle R. Stone

**Affiliations:** 1 College of Kinesiology, University of Saskatchewan, Saskatoon, SK S7N 0W9, Canada; natalie.houser@usask.ca; 2 Healthy Populations Institute, Dalhousie University, Halifax, NS B3H 4R2, Canada; janescawley@gmail.com (J.C.); daniel.rainham@dal.ca (D.R.); sara.kirk@dal.ca (S.F.L.K.); 3 Department of Human Kinetics, St. Francis Xavier University, Antigonish, NS B2G 2W5, Canada; akolen@stfx.ca; 4 Department of Earth and Environmental Sciences, Dalhousie University, Halifax, NS B3H 4R2, Canada; 5 School of Health and Human Performance, Dalhousie University, Halifax, NS B3H 4R2, Canada; laurene.rehman@dal.ca; 6 Child and Youth Study, Mount Saint Vincent University, Halifax, NS B3M 2J6, Canada; joan.turner@msvu.ca

**Keywords:** physical activity, early childhood, preschool, parents, educators, child care centre

## Abstract

BACKGROUND: The Physical Literacy in the Early Years (PLEY) intervention is a randomized mixed-methods controlled trial focused on embedding loose parts materials into the outdoor play spaces of regulated child care centres across Nova Scotia. The aim is to evaluate the efficacy of the PLEY intervention versus standard regulated childcare practice in influencing thoughts and behaviors of children, parents, and educators. METHODS: Participating early child care centres (n = 19) were randomly assigned to intervention or control sites. Intervention sites received loose parts kits at the beginning of the project while control sites received kits upon project completion. The kits included items such as rocks, tree cookies, balls, wood planks, tubes, tires, ropes, and pulleys. Children (n = 183 at baseline) had their physical activity (accelerometers) and movement skills (TGMD-3 and PGMQ) measured before and after the intervention. All centres provided responses to environmental surveys (Go NAP SACC and Site Context Questionnaire), and educators in intervention sites participated in focus group and photovoice sessions. Educators were also provided with a full day professional development opportunity (plus ongoing mentoring) focused on physical activity, physical literacy, outdoor play, risk-taking, and loose parts. Parents participated in an interview addressing active outdoor play, physical literacy, and attitudes towards risk taking during play. DISCUSSION: This study will provide a better understanding of how integrating loose parts materials into outdoor play spaces impacts children’s health, and the impact on educator and parent attitudes, beliefs, and understanding around physical literacy, active outdoor play and risk-taking during play.

## 1. Introduction

Physical activity (PA) participation in the early years (age 0–4 years) and in school-aged children and youth (age 5–17 years) is associated with a wide range of physiological, psychological and socio-emotional health benefits that can track into adulthood, and importantly, contribute to a decreased risk of chronic disease [1]. Several systematic reviews demonstrate favorable associations of PA with motor skill development, adiposity, fitness, bone and skeletal health, psychosocial and cardio-metabolic health, and cognitive development [1,2,3,4]. A recent review, including 96 studies representing 71,291 children from 36 countries, suggested the more PA, the better (in terms of health benefits) for young children (age 0-4 years), with moderate-to-vigorous physical activity (MVPA) and total physical activity consistently favorably associated with health benefits [4]. Limiting sedentary behavior (SB), particularly screen time [5] and attaining optimal levels of sleep (quality and quantity) [6] are also critical for healthy growth and development during the early years.

While these movement behaviors are often studied in isolation in relation to health outcomes in childhood, emerging evidence is showing that they interact with one another to impact children’s health [7,8,9]. Kuzik and colleagues’ recent systematic review of the relationships between combinations of movement behaviors and health indicators in the early years (age 0–4 years) illustrated that the most ideal combination of PA and SB were favorably associated with motor development and fitness in preschoolers. Comprehensive 24-h movement guidelines are now available for the early years [10] and school-aged children and youth [11]. These guidelines outline a whole day approach to movement and highlight the importance of maintaining health behaviors (PA, sleep and limited SB) across a child’s day. According to these guidelines, preschool-aged children (age 3–4 years) should spend at least 180 min per day engaged in a variety of PAs (structured and unstructured), of which at least 60 min is energetic play; with more PA being better [10]. Preschoolers should not be restrained for more than one hour at a time or sit for extended periods; sedentary screen time should be no more than one hour/day (less is better); and when sedentary, engaging in reading and storytelling is encouraged. It is also recommended that preschool-aged children obtain 10 to 13 h of quality sleep a night, maintaining consistent bed- and wake-times [10].

Despite this evidence, Canadian preschoolers spend very little of their waking hours physically active, and more of their time sedentary. Analyses of Canadian Health Measures Survey data (Cycles 2–4: 2009–2011; 2012–2013; 2014–2015), representing 803 children aged 3 to 4 years, revealed that participants were physically active for 4.6 h per day, on average, with approximately one hour spent in MVPA [8]. Just 12.7% of preschoolers meet the 24-h movement guidelines, with a high proportion (61.8%, and 83.9%) meeting the PA and sleep recommendations, respectively, and a minority (24.4%) meeting the screen time recommendations (3.3% of preschoolers met none of the recommendations). Together, these findings, and previous reports [12], suggest that the majority of Canadian children aged 3 to 5 years are spending too much of their day sedentary (particularly in screen-time behaviors) displacing time that could otherwise be spent physically active.

Understanding key influencers of preschoolers’ movement behaviors is crucial to determine how best to support opportunities to be physically active in the early years. Several systematic reviews of correlates and determinants of PA during the early years have identified time spent outdoors [13,14], the preschool setting [15], and motor skill development [2,4] to have a favorable impact on preschoolers’ PA behaviors. Considering the impact that these variables can have within an early years environment, there is an opportunity to emphasize movement exploration and active play in young children, particularly in the outdoor environment.

There is considerable evidence to suggest that children’s opportunities to engage in outdoor play have steadily declined over the past several generations [16,17]. Children spend less time now outside than their parents did [18,19] and there has been a shift from unstructured and unsupervised outdoor play to structured and supervised activities (mostly indoors) [20,21]. The benefits of active outdoor play to children’s healthy growth and development are well established [16,17] with increased attention placed recently on increasing children’s participation in it. The ParticipACTION Position Statement on Active Outdoor Play describes that “access to active play in nature and outdoors- with its risks- is essential for healthy development” and recommends, “increasing children’s opportunities for self-directed play outdoors in all settings—at home, at school, at child care, the community, and nature.” [22].

Social and physical environments have an important influence on the PA, health, and wellbeing of children. Early childhood education and care services can provide social and physical environments that support children’s PA and outdoor play opportunities. In child care centres specifically PA participation is frequently reported to be low [23]. A Canadian study including preschoolers revealed that children spent on average just 1.5 min per hour in MVPA, and 40.6 min per hour sedentary [24]. Given that Canadian preschoolers spend an average of 29 h a week in childcare [25], this environment is critical for encouraging active behaviors. PA interventions in child care settings have demonstrated varying levels of success. Some interventions significantly improved PA behaviors during the preschool day [26]; however, these improvements were not maintained in 6-month or 12-month follow-ups [23]. As such, conducting research in child care settings provides ample opportunity to encourage sustainable change. These findings may suggest that future interventions need to focus on more than just PA, encouraging a more holistic approach to incorporate broader constructs like physical literacy.

The preschool years are seen as opportune for developing fundamental movement skills (FMS) due to children in this age group experiencing rapid brain growth and neuromuscular maturation, as well as high levels of perceived competence [27]. FMS are one component of a more holistic concept, physical literacy, the “motivation, confidence, physical competence, knowledge, and understanding to value and take responsibility for engagement in physical activities for life” [28,29]. Physical literacy development during the early years is critical for establishing lifelong PA participation [30]. This term, and exploration of components of physical literacy (PL), has started to gain traction in early years’ health-related research [31,32], with increasing interest in exploring settings in which physical literacy development can be supported. As there are limited measurement tools currently validated for use in the preschool age group, this study will seek a more holistic approach to physical literacy through the measurement of movement skills and physical literacy, but also through data collection with parents and educators regarding child behaviors, motivations, and confidence toward PA.

To further enhance physical literacy development, it is encouraged that children are provided with the opportunity to explore new movements and environments. One way to encourage this exploration is through active, outdoor play. Children’s opportunity for unstructured and risky play, as discussed in the 2015 ParticipACTION report card, has been limited given the changes to outdoor play spaces and reduced opportunity overall for outdoor play [33]. Incorporating loose parts into the outdoor play environment may pique children’s curiosity and exploration of movement. Loose parts are described as materials that can be moved and manipulated in various ways to encourage creative thinking, and the opportunity for exploration of their environment [34]. These materials can be from the natural environment (leaves, sticks, etc.) or synthetic (rope, buckets, etc.), and can used in the indoor or outdoor environment.

Loose parts provide opportunities for children to enhance their creativity, collaborative behaviors, and cognitive functioning [35]. An important consideration with loose parts is that the materials be open-ended, to allow for unstructured child-led play, and for children to make use of these materials any way they choose. Although the concept of loose parts has existed for many years [34], to the authors’ knowledge, no evidence exists on the efficacy of integrating loose parts materials in centres’ outdoor spaces as a means of improving children’s physical literacy and increasing PA and active outdoor play [36]. Considering the exploratory and open-ended nature of loose parts materials, it is possible that these materials could influence the basic aspects associated with physical literacy, including movement competence, confidence and motivation, and daily behaviors, increasing the likelihood of lifelong PA participation [30].

### Study Objectives

The objectives of this research were to evaluate the efficacy of the loose parts intervention versus outdoor play practice in standard early child care settings to:

(1) improve children’s physical literacy, and increase time in PA and active outdoor play;
(2) improve educators’ attitudes, beliefs, perceived competency, and intentions towards incorporating the intervention into practice;
(3) increase parents’ and educators’ understanding of play in child health and development.

## 2. Experimental Design

### 2.1. The Intervention

**Figure 1:**
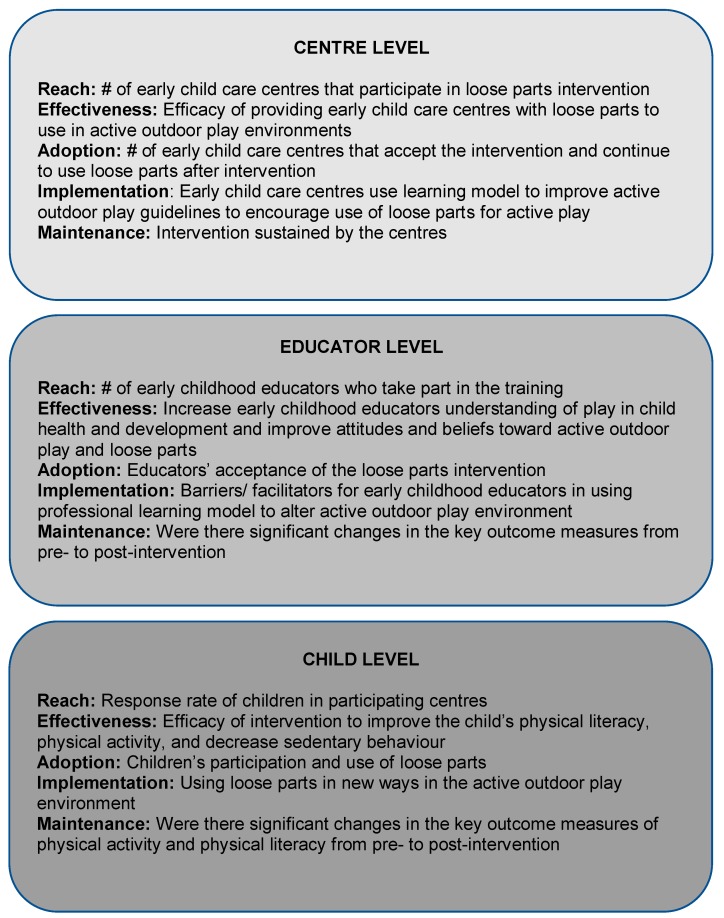
RE-AIM framework outline for the Physical Literacy in the Early Years (PLEY) project.

The Physical Literacy in the Early Years (PLEY) project is a randomized, mixed-methods, controlled trial focused on improving physical literacy, PA and active outdoor play in Nova Scotia preschoolers aged 3 to 5 years, through the integration of loose parts materials into outdoor play spaces in regulated child care centres. The PLEY project uses a Socio-Ecological approach [37] to address multiple levels of influence such as the intrapersonal, interpersonal, organizational, community, physical environment, and political levels. An internationally recognized socio-ecological framework, the RE-AIM framework (reach, effectiveness, adoption, implementation, and maintenance) [38], is used to examine research translation and dissemination, and evaluate the intervention. The RE-AIM framework evaluates the ‘reach’ to the target population; the ‘effectiveness’ of the intervention; the extent of ‘adoption’ of the intervention in child care centres; implementation; and ‘maintenance’ of the intervention effects. The RE-AIM framework for centre, educator and child levels is highlighted in Figure 1. This framework approach allows for the possibility to inform current policy and practices in Nova Scotia child care centres at the different levels impacted. This trial was retrospectively registered in October 2017 with BioMed Central under the trial registration number ISRCTN14058106.

The PLEY project was developed using an interdisciplinary, multi-sectoral partnership of researchers, practitioners (early childhood educators), government and policy-makers. The practitioner lens is critical to the partnership, as it provides a deeper understanding of the typical processes that take place in child care centres on a day-to-day basis and gives insight into optimal ways in which the intervention and research can be successfully conducted.

**Figure 2:**
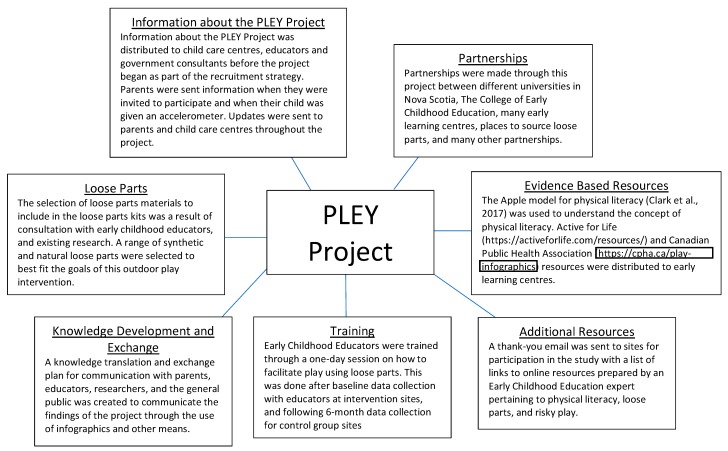
PLEY project components

The goal of the PLEY project is to encourage and enable early childhood educators to integrate loose parts materials into child care centres’ outdoor play spaces as a means of improving preschoolers’ physical literacy and increasing time in PA and active outdoor play. The intervention is composed of seven components, which are detailed in Figure 2.

### 2.2. Methods and Design

The primary aim of this randomized, mixed-methods controlled study was to evaluate the efficacy of a loose parts intervention versus standard early years settings’ practice to improve children’s physical literacy, and increase time in PA and active outdoor play. The intervention spanned a period of six to eight months. Funding for the project was secured in November 2015, and the study protocol received ethical approval from the Research Ethics Board (REB 2016-3924) in July 2016. Data collection from the 19 participating sites (intervention: n = 11; control: n = 8) took place from April, 2016- September, 2018. This study was registered as a trial with BioMed Central (ID# ISRCTN14058106) in October 2017 and can be found at http://www.isrctn.com/ISRCTN14058106.

#### 2.2.1. Study Design

A total of nineteen (19) child care centres across Nova Scotia (spread across over 240 km) were randomly allocated to either the intervention (n = 11) or control (n = 8) group. The study took a staggered approach to the recruitment of child care centres. Sixteen sites (intervention: n = 8; control: n = 8) were recruited in November/December of 2016 (Phase 1). An additional three intervention sites were recruited in November of 2017, to account for the drop-out of 1 centre in October of 2017, and participant (child) withdrawal. This study adheres to the CONSORT guidelines for randomized trials. Quantitative and qualitative data were collected at baseline, 3 months, and 6 months post-intervention. Quantitative data were collected from 183 participants at baseline through movement assessments and accelerometry. The same assessments occurred at 3 months post intervention and 6 months post intervention. Centres in the intervention group were provided with a loose parts kit to incorporate in their active outdoor play. The other centres were asked to maintain current scheduling of outdoor play and related activities and received a loose parts kit at the end of the intervention. The loose parts kits for the PLEY project intervention were intended for the outdoor environment and included: buckets and lids (variety of shapes and sizes), rope and pulley, tree cookies, milk crates, a package of hose tube, 20+ balls; a variety of sizes and weights, wood pieces, a bread tray, large cardboard tubes, funnels of different sizes, a tarp, 5′ planks, 5′ PVC tubing (4” and 2” diameter), rocks, and tires. The contents of the loose parts kits were determined based on consultation with the existing literature and discussions with early childhood educators to select natural and synthetic loose parts that best suited the purposes of this project.

#### 2.2.2. Target Population and Sampling

Recruitment commenced with a general inquiry of interest that was sent to child care centres across Nova Scotia that served children between the ages of 3 and 5 years with an enrolment greater than 20 children. The Department of Education and Early Childhood Development was instrumental in disseminating information about the research project to regulated child care centres throughout the province. Allowing for non-participation rates of 20%, we anticipated participation by 180 children across the intervention or control groups. As seen in previous research, this sample size of child participants is sufficient to have an 80% chance of detecting a 10% difference in physical literacy between the composite scores of the intervention and control group at the 5% significance level [39]. This sample should be sufficient to detect moderate between-group effects in fundamental movement skills [39]. Intra-class correlation (ICC) estimations were determined based on a similar study on preschool-aged children, which calculated an ICC of 0.02 [32]. Based on similar studies, we estimate moderate effect size on fundamental movement skills [40], and small-to-moderate effect size on physical activity [41].

**Figure 3:**
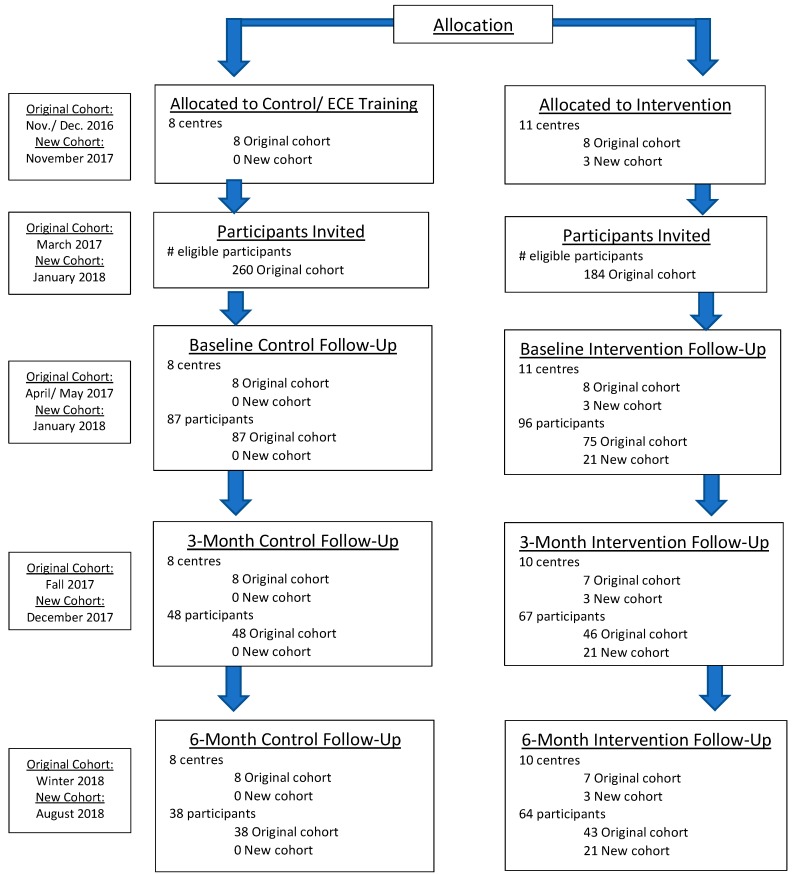
Flow diagram of participant numbers (children).

Initially, 21 child care centres in Nova Scotia expressed interest in participating in the study and met study criteria. A visit was scheduled with the centres to meet with the director, further discuss the project, discuss and complete a survey, and view/photograph their designated outdoor play space and adjacent spaces if applicable. Two sites were excluded because they were advanced in their use of loose parts for outdoor play. Three other sites were excluded due to a low response rate to the invitation to participate. For efficacy, the minimum required for a centre to be involved was 10 children, and these centres only received parental consent for 3–5 children. This resulted in a final number of 16 centres in the original cohort, and 3 centres in the new cohort participating in the study. All children between the ages of 3 and 5 years attending the involved child care centres were eligible to participate in the intervention or control groups. Only children whose parents provided informed written consent were formally assessed. At the time of data collection, children also had to provide assent to participate in the assessment (e.g., agreeing to wear the accelerometer). Once the initial recruitment phase was complete and it was determined which centres met all the inclusion criteria (16 sites total), baseline data collection for children (movement assessment and accelerometer measurements) was completed in May 2017. The participating centres were then randomly assigned to the control or intervention group through computer based random number selections, based on rural and urban locations dispersed between the two groups. The full breakdown of number of centres and participants can be seen in Figure 3.

## 3. Procedure

### 3.1. Children

#### 3.1.1. Demographic and Body Composition

Demographic data, including age and sex, and physical characteristics, including height and weight, were taken by trained personnel at child care centres. Height was assessed using a portable stadiometer (SECA, Hamburg, Germany) and taken to the nearest 0.1 cm. Weight was assessed using a digital scale (A&D Medical, Milpitas, CA, USA) and taken to the nearest 0.1 kg. Children’s height and weight were measured with children wearing light clothing and no footwear. The height and weight of each child were used to determine Body Mass Index (kg/m^2^), which will be interpreted using z-scores. Data were collected at baseline, and at 3- and 6-months post-intervention.

#### 3.1.2. Physical Literacy

The Test of Gross Motor Development-3 (TGMD-3) was used to evaluate children’s fundamental movement skills (FMS) [42]. The TGMD-3 is a validated tool that measures gross motor ability development for children aged 3 to 11 years through a qualitative process-oriented approach comparing each child’s results to pre-determined standardized norms. The test is ideal for evaluating the success of motor skill interventions in this age group. A sum of all locomotor skills (run, skip, slide, gallop, hop, and jump) and object control skills (overhand throw, underhand throw, catch, dribble, kick, one-hand strike, two-hand strike) was determined, and used to calculate a total gross motor score (total FMS). Balance was assessed using the Preschooler Gross Motor Quality Scale (PGMQ), a validated tool which includes 4 balance measurements (single leg standing, tandem standing, walking line forward, and walking line backward) [43]. The assessment took a total of 15 to 20 min to complete. Each trained researcher/evaluator assessed one child at a time, going through the multiple performance criteria for each skill, and provided a score. The evaluator first demonstrated how to correctly perform the skill to the child, then asked the child to perform the skill. The children were given one practice trial, followed by two scored test trials.

#### 3.1.3. Physical Activity

Children’s PA was measured using accelerometry (ActiGraph wGT3X+; ActiGraph, LLC, Pensacola, FL, USA) during waking hours for nine consecutive days. In order to improve compliance and ensure data quality, parents were given an instruction sheet that explained how to attach the accelerometer over their child’s right hip and when the device was to be removed (night-time sleep, bathing/swimming). Parents and educators were also informed of the importance of consistent accelerometer wear to provide typical physical activity and sedentary behavior patterns. Accelerometer data were reduced and analyzed using ActiLife (Version 6). To improve comparability of data, accelerometer data collection and reduction decisions were consistent with a previous study of Canadian preschoolers [44]. Data were collected in 15 s epochs. Non-wear time was defined as ≥20 min of consecutive zero counts [44]. To be included in analyses, participants were required to have ≥4 days with ≥6 h of wear time each day [45]. A weekend day was not necessary for inclusion. Sedentary time was defined as ≤100 counts/min, light physical activity (LPA) as 100-1679 counts/min, and MVPA as ≥1680 counts/min [46].

### 3.2. Educators

#### 3.2.1. Go NAP SACC Outdoor Play and Learning Self-Assessment Instrument

The Nutrition and Physical Activity Self-Assessment for Child Care (Go NAP SACC) is an outdoor play and learning self-assessment tool that captures information on outdoor play time, outdoor play environment, education and professional development, and policy [47]. This tool was used to assess the outdoor environments of participating child care centres at baseline, and was completed by centre directors (CD) and/or early childhood educators (ECE). A total of 19 centers completed the survey; 16 from the original cohort, and 3 from the new cohort.

#### 3.2.2. Site Context Questionnaire

The site context questionnaire was developed to gather contextual information that was not captured through the Go NAP SACC instrument on outdoor play time, outdoor play equipment, outdoor play environment, centre policies, and loose parts materials. Specific questions were taken from the Environment and Policy Assessment and Observation (EPAO) tool [48], and others have been modified in a way that requires reflection rather than direct observation. The questionnaire was pilot tested with the former executive director of the Nova Scotia College of Early Childhood Education, to ensure the questions were appropriate for the intended audience. The questionnaire was completed by site directors, educators, and other centre staff during the intervention, and took approximately 20 min to complete.

#### 3.2.3. Educator Training Session

The full day educator training session was facilitated with the intervention sites after the baseline movement assessments and accelerometer measurements were completed. Seven of the eight intervention sites in the original cohort attended: 15 participants (13 ECEs and 2 CDs) took part in the training session; and an alternative training session was subsequently delivered to the one site (educator, assistant director and special needs coordinator) unable to attend the regular training session. In the new cohort, two training sessions were offered; 24 attended the first session, 21 attended the second session. Educators (preschool classrooms), support staff and centre directors were introduced to the project team, the goals, the research methodology and most importantly their role. Participants focused on the key components of the PLEY project including physical literacy and fundamental movement skills, active/outdoor play and loose parts, and the benefits of risk-taking in children’s play. This session also allowed for discussion among attendees from different child care centres to share experiences and stories that further informed the session. The training session was delivered through presentations and hands-on activities designed to promote the use of loose parts for active outdoor play, increase educator understanding of the importance of PA for young children, support observation and documentation, and explain the concepts of physical literacy. Educators had the opportunity to evaluate and describe photographs of children exploring loose parts and to “play” with the loose parts in an outdoor space. The loose parts kits were distributed to the intervention sites upon the completion of the training session. The session provided 6.5 professional development credit hours for the educators.

#### 3.2.4. Photo Documentation and Elicitation, and Focus Groups

Photo documentation and elicitation, and focus group sessions, were conducted at the 3-month and 6-month stage of the loose parts intervention. Photo elicitation involved participants photographing something specific prior to meeting with the research team for discussion of their photos; photo elicitation has previously been used to explore children’s health, physical activity, school environments, play and recreation [49,50,51,52]. This strategy/process for observing, documenting, and sharing stories of children playing with loose parts was designed to highlight and support the educators’ role in the intervention. The strategy, introduced at the training session, involved taking photographs accompanied by a documentation form focused on physical literacy, fundamental movement skills, and the educators’ role. Educators were asked to photograph, document, and submit examples of loose parts play that “caught their attention” as often as they could. Feedback, assistance and encouragement were made available throughout the intervention.

At the 3-month stage, all intervention participants were invited to attend a photo elicitation sharing and focus group activity to explore and share their experiences and to document changes they noted in their attitudes, beliefs, perceived competency, and intentions towards incorporating loose parts into the outdoor play environment. Each attendee (9 from original cohort; 15 from new cohort) was asked to bring a photo with documentation that demonstrated compelling evidence of how loose parts play supports physical literacy. The participants were organized into small groups (3 to 4 per group) to ensure that there would be opportunities for sharing between centres. Each focus group had a facilitator and a note-taker, and was audio-recorded. At the 6-month focus group sessions, both intervention and control centres were invited to participate. At the completion of these focus group sessions, the control groups received their loose parts kits.

During the photo elicitation sharing activity the educators described the play patterns of the children in the photos and highlighted how active play with loose parts helped children learn fundamental movement skills which supports their physical literacy development. The stories provided more context and allowed for better understanding of play in healthy child development. It was a catalyst for in-depth discussions and served to trigger comments from other participants about their own experiences. This activity was followed by a series of focus group questions divided into several categories: outdoor active play, loose parts, risk-taking, policies, and challenges/benefits of the intervention. These focus groups, which lasted approximately 45 to 60 min, allowed for more in-depth exploration of what made it challenging, or what assisted them in using the loose parts in their daily activities. On-site focus groups were facilitated for the two child care centres that did not attend the original session (4 educators).

### 3.3. Parents

#### 3.3.1. Parent Survey

Parents completed a 10 to 15-min survey at baseline with questions pertaining to themselves and their child, to better understand how children engage in active outdoor play and the factors potentially involved. The first section of the survey gathered information on parent demographics, physical activity participation (parent and child), sleep (child), sedentary behavior (parent), and parent perceptions of their child’s physical literacy. Parent perceptions concerning the level of risk associated with children’s physical activity/play behavior, and affordances for risk taking during physical activity/play, were also assessed. A total of 85 families completed the parent survey; 76 from the original cohort, and 9 from the new cohort.

#### 3.3.2. Parent Interviews

At the end of the 6-month intervention, a subsample of parents in the intervention and control groups was invited to participate in parent interviews. Each director was asked to recommend two to four parents who might be willing to continue their involvement in the PLEY project and these parents were then invited to participate. The interviews were conducted by a facilitator and audio-recorded, with the assistance of a note-taker at 10 sites with 18 parents (individually or in small groups). The semi-structured interviews asked questions related to a typical day for their child both indoors and outdoors with a focus on active outdoor play, and explored the who, what, when, and where, of active outdoor play. Interviewers probed whether changing seasons and inclement weather influenced play decision-making. There was an overall interest in discovering what the parents learned about the project, about physical literacy and about the role of active outdoor play in their child’s healthy growth and development through participation in the PLEY project. Finally, there was an interest in learning about parent attitudes towards risk taking during play and seeing if those attitudes have an influence on parenting behaviors.

### 3.4. Data Analysis

Descriptive statistics will be used to describe study participants (children, educators, parents) and participating child care centres. Linear mixed models with a random effect for centre will be used to determine if children exposed to the PLEY intervention had greater increases in physical literacy, PA, and time in active outdoor play, compared to children in the usual care group (control group). The model will include variables from the children’s TGMD-3 scores, PGMQ scores, and physical activity (as determined by accelerometry). The analysis will be approached from an intention-to-treat strategy. One of the primary outcome measures will be physical activity differences between the groups, while secondary outcomes include height, weight, BMI, and age. Additional analyses of the variables may include an analysis of covariance (ANCOVA) to determine differences in TGMD and PGMQ scores, with similar secondary outcomes of height, weight, BMI, and age, and again including a random effect to control for the clustered design. Missing data will be controlled for within the selected models and analyses. Significance level for these analyses will be set a *p* < 0.05.

Educator focus group data, and parent interview data, will be explored using qualitative analyses, to determine whether the intervention improved educators’ attitudes, beliefs, perceived competency, and intentions towards incorporating the intervention into practice; and increased parents’ and educators’ understanding of play in child health and development. These qualitative data will be transcribed and analyzed using NVIVO software to identify emerging themes. Children’s physical literacy and PA data will be examined in comparison with parent survey and interview responses, and educator survey and focus group responses, using a mixed methods approach. Data from the RE-AIM framework analysis and qualitative data emerging from the photo elicitation focus groups with intervention sites will determine the effectiveness of the intervention to achieve desired outcomes. The mixed methods approach will provide a more holistic understanding of the impact of the PLEY intervention on children’s physical literacy. Although the child measures only include some domains of physical literacy (e.g., physical competence and daily behavior) other aspects of the study including educator focus groups allow us to obtain data on the other components of physical literacy.

## 4. Discussion

As the purpose of this paper is to describe the purpose, study design and methodology of the PLEY loose parts intervention, no results are included in this paper. It is hypothesized that children in centres exposed to the loose parts intervention will experience greater increases in physical literacy, PA and active outdoor play than the usual practice centres. Previous studies similar to this have reported changes (mean(95%CI)) of −0.2(−1.7 to 1.3) in physical activity between groups [53], and 2.5 (−1.7 to 6.7) in the sum of scores for fundamental movement skills between groups [40]. It is also hypothesized that parents and educators involved in the intervention centres will gain a greater understanding of physical literacy, PA, and active outdoor play. It is hypothesized that educators will be exposed to an increase of risk-taking behaviors with the introduction of loose parts into outdoor play environments and that they will begin to recognize their children’s ability to assess risk independently. Finally, it is also hypothesized that the loose parts intervention has the potential to clarify the role of the educator as a guide, a mentor and a co-player in outdoor environments.

This study consists of various strengths and limitations to be acknowledged. The major strength of this study lies in the uniqueness of an outdoor play intervention with the use of loose parts, the inclusion of urban, suburban, and rural regions of Nova Scotia, and in the measurement of child, parent and educator components. The limitations of this study include the dropout rate of children, which resulted in a lower sample size during the post-testing, and that not all components of physical literacy were measured as part of this study, given the age of the child.
